# Special Issue “Diagnosis and Monitoring of Kidney Disease: From Biology to Biomarkers”

**DOI:** 10.3390/ijms27208942

**Published:** 2026-10-09

**Authors:** Susana Coimbra, Flávio Reis, Alice Santos-Silva

**Affiliations:** 1 UCIBIO i4HB, Faculdade de Farmácia, Universidade do Porto, Rua Jorge Viterbo 228, 4050-313 Porto, Portugal; assilva@ff.up.pt; 2 UCIBIO i4HB, Translational Toxicology Research Laboratory, CESPU University, Avenida Central de Gandra 1317, 4585-116 Gandra, Portugal; 3 Institute of Pharmacology & Experimental Therapeutics & Coimbra Institute for Clinical and Biomedical Research (iCBR), Faculty of Medicine, University of Coimbra, Azinhaga de Santa Comba, Celas, 3000-548 Coimbra, Portugal; freis@fmed.uc.pt; 4 CIBB—Center for Innovative Biomedicine and Biotechnology, University of Coimbra, Rua Larga, Edifício FMUC, Piso 1, 3004-504 Coimbra, Portugal; 5 Clinical Academic Center of Coimbra (CACC), Praceta Professor Mota Pinto, 3004-561 Coimbra, Portugal

Kidney diseases, particularly chronic kidney disease (CKD), represent a major and growing health challenge, imposing a substantial economic burden and significantly compromising patients’ quality of life. Current estimates indicate that CKD affects approximately 10% of the global population and is projected to become the fifth leading cause of mortality worldwide by 2040 [1]. Because CKD is typically asymptomatic in its early stages, many individuals remain undiagnosed until the disease has progressed [2]. Acute kidney injury (AKI), a clinical syndrome characterized by a sudden decline in kidney function, affects an estimated 13 million people worldwide each year and is associated with increased morbidity, mortality, and a heightened risk of developing or accelerating CKD [3,4].

A comprehensive understanding of the biology of kidney diseases, particularly the biomolecules and molecular mechanisms underlying disease onset and progression, is essential for the identification of biomarkers for early diagnosis, as well as for improving disease monitoring and management. Elucidating the molecular signaling pathways involved in key components of CKD pathophysiology, including inflammation, oxidative stress, and renal fibrosis, is crucial for facilitating earlier diagnosis, improving disease monitoring, and discovering more effective therapeutic strategies.

Conventional biomarkers of kidney function often become altered only after substantial renal impairment has occurred. Early diagnosis is essential to improve clinical outcomes, delay disease progression, and reduce the risk of renal complications or of other associated comorbidities, namely cardiovascular diseases.

Identifying sensitive and reliable biomarkers capable of detecting early renal injury and predicting kidney disease progression remains a significant research priority.

This Special Issue comprises six manuscripts (Figure 1) that highlight the growing value of recent advances in identifying molecular biomarkers in kidney diseases, particularly for two complementary purposes—improving early diagnosis and risk prediction and enhancing the monitoring of disease progression and therapeutic response. By providing us with a better understanding of the molecular mechanisms that drive kidney injury, they contribute to pave the way for more precise and personalized patient management.

In a clinical study involving 435 participants with CKD who completed 14 weeks of treatment in the phase II trial with vicadrostat, a selective aldosterone synthase inhibitor, administered with or without empagliflozin, a sodium–glucose cotransporter 2 inhibitor, Delic et al. demonstrated that this combined therapy elicited broader molecular effects than vicadrostat monotherapy. These enhanced effects were likely mediated through the modulation of fibrotic and immunoregulatory pathways via changes in urinary extracellular vesicle microRNAs, independently of the participants’ diabetes status. These findings provide a mechanistic explanation for the greater reductions in albuminuria observed in the clinical trial. Furthermore, urinary extracellular vesicle microRNAs were identified as promising non-invasive biomarkers for monitoring the response to aldosterone synthase inhibition, given their ability to reflect key molecular processes involved in kidney injury and disease progression. This study highlights the potential of microRNA profiles as valuable biomarkers for disease activity and treatment response in CKD.

Kidney transplantation remains the optimal renal replacement therapy for patients with end-stage renal disease. Living-donor transplantation provides superior graft survival and long-term outcomes compared with deceased-donor transplantation [5]. However, understanding the biological consequences of living kidney donation is essential to ensure donor safety, particularly regarding potential cardiovascular risks associated with reduced renal mass [6]. In this context, Torres et al. investigated the impact of changes in kidney function on endothelial activation and inflammatory pathways in 50 pairs of living kidney donors and transplant recipients, in a non-interventional, prospective, multicenter, longitudinal study. Their findings provide valuable insights into the molecular mechanisms linking renal function with systemic vascular alterations, showing that reduced kidney function in living donors was associated with increased endothelial activation and inflammatory responses, whereas restoration of renal function in transplant recipients following transplantation reversed several of these abnormalities. Importantly, this study highlights the potential of circulating biomarkers, including soluble vascular cell adhesion molecule-1 and soluble tumor necrosis factor receptor, to monitor the biological changes associated with kidney function. Although no adverse clinical outcomes were observed during the one-year follow-up period, these findings emphasize the importance of long-term monitoring and further investigation of biomarkers capable of improving risk assessment and personalized management of kidney donors and recipients.

Episodes of AKI can trigger persistent renal inflammation, endothelial dysfunction, maladaptive repair, and fibrosis, potentially leading to incomplete recovery and long-term loss of kidney function [7]. In this framework, Martin et al. evaluated the performance of proenkephalin A 119–159 (PENK) as a biomarker of kidney function and AKI in routine clinical practice, based on almost 18,000 PENK measurements from 4169 patients. Their one-year evaluation suggested that PENK is a practical and clinically useful early biomarker for assessing kidney function in critically ill patients. Its main strengths include earlier detection of declining glomerular filtration, prediction of AKI development and need for renal replacement therapy, identification of patients at increased risk of persistent renal dysfunction, and the provision of complementary information to conventional biomarkers.

Exposure to toxic elements, such as cadmium and mercury, is known to induce renal tubular injury [8,9] and may contribute to the development of AKI, particularly following high-level exposure. Chronic or repeated contact with these nephrotoxic elements may promote long-term renal dysfunction and increase the risk of CKD progression. Additionally, an elevated copper-to-zinc (Cu/Zn) ratio has been identified as a potential biomarker associated with increased oxidative stress, inflammation, and accelerated decline in kidney function [10]. By investigating the association between urinary concentrations of trace elements and biomarkers of kidney injury in 914 healthy adolescents (11–18 years old), Ortega-Romero et al. demonstrated that cadmium exposure was associated with tubular damage in this young, healthy population, even at relatively low environmental exposure levels. Furthermore, the Cu/Zn ratio correlated with markers of kidney injury among individuals with low cadmium exposure, suggesting that a trace element imbalance may be a potential predictor of renal function decline.

Lee et al. summarize current evidence on biomarkers that may improve the diagnosis, risk stratification, and prevention of contrast-induced nephropathy, also referred to as contrast-associated acute kidney injury (CA-AKI). Their review highlights neutrophil gelatinase-associated lipocalin (NGAL) as one of the most promising early biomarkers; cystatin C and beta-2 microglobulin as valuable biomarkers of glomerular filtration; and kidney injury molecule-1 and interleukin-18 as useful biomarkers of tubular injury. Additionally, several markers, such as L-type fatty acid-binding protein, tissue inhibitor of metalloproteinases 2, insulin-like growth factor-binding protein 7, and PENK, are considered as emerging CA-AKI biomarkers. The authors conclude that future approaches to CA-AKI management will likely rely on panels of complementary biomarkers rather than on a single one; these panels could be identified and validated by applying artificial intelligence and machine learning technologies.

Maslyennikov et al.’s review, on the other hand, summarizes the current understanding of the relationship between minimal change disease (MCD) and focal segmental glomerulosclerosis (FSGS), with particular emphasis on proteomic technologies. The authors propose that MCD and FSGS should not be considered as entirely distinct entities, but rather as part of a spectrum of podocyte disorders sharing overlapping molecular mechanisms. Podocyte injury is identified as the central pathogenic event, with immune dysregulation also playing a key role in these conditions’ development. Proteomics, particularly urinary proteomics, has emerged as a promising approach for biomarker discovery. However, despite its potential, the authors emphasize that thus far, no proteomic biomarker has undergone sufficient validation for routine clinical use. A sequential strategy for proteomic biomarker development is proposed, in which tissue proteomics could initially identify proteins with altered expression profiles, reflecting underlying disease mechanisms, followed by validation of these candidates in urine or plasma to enable the development of diagnostic and prognostic biomarker-based approaches.

Collectively, these studies underscore recent advances in the discovery and validation of biomarkers for kidney diseases, with a primary focus on improving early diagnosis and risk prediction, followed by the optimization of disease monitoring and therapeutic response assessment. By linking molecular mechanisms involved in kidney injury, inflammation, endothelial dysfunction, fibrosis, and immune dysregulation to measurable biological biomarkers, these studies contribute to the development of more precise and personalized strategies for the management of kidney diseases.

AI—artificial intelligence; AKI—acute kidney injury; CA-AKI—contrast-associated AKI; Cu—copper; EV—extracellular vesicle; FSGS—focal segmental glomerulosclerosis; GFR—glomerular filtration rate; IGFBP7—insulin-like growth factor-binding protein 7; IL—interleukin; KIM1—kidney injury molecule-1; MCD—minimal change disease; miRNAs—microRNAs; ML—machine learning; NGAL—neutrophil gelatinase-associated lipocalin; PENK—proenkephalin A 119–159; sVCAM—soluble vascular cell adhesion molecule; sTNFR—soluble tumor necrosis factor receptor; Zn—zinc.

## Figures and Tables

**Figure 1:**
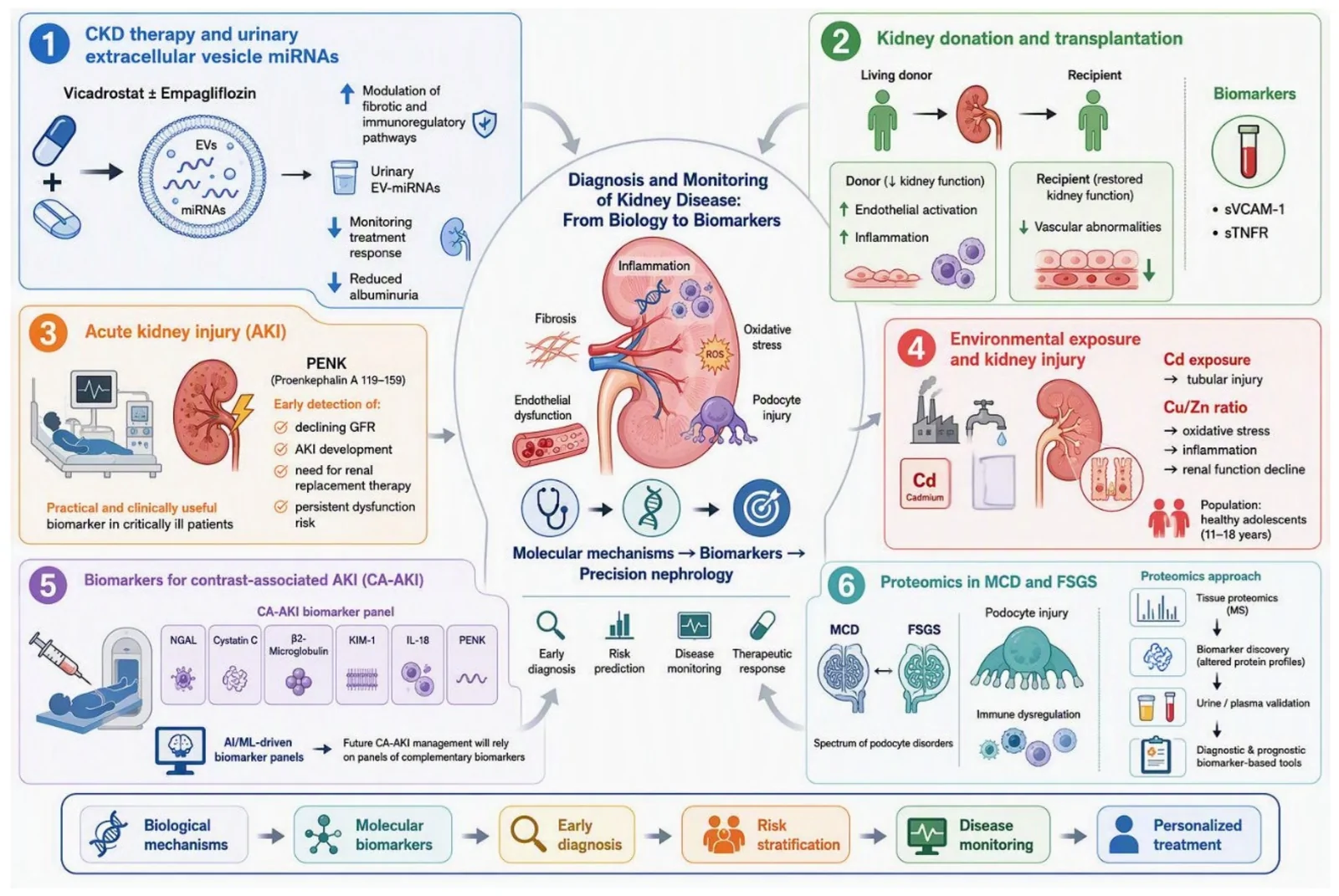
A schematic representation of the main findings reported in the Special Issue “Diagnosis and Monitoring of Kidney Disease: From Biology to Biomarkers”. The visual layout and graphical elements were developed with the assistance of Microsoft Copilot and subsequently reviewed and edited by the authors.

## References

[1] Li P.K., Garcia-Garcia G., Lui S.F., Andreoli S., Fung W.W., Hradsky A., Kumaraswami L., Liakopoulos V., Rakhimova Z., Saadi G. (2020). Kidney health for everyone everywhere-from prevention to detection and equitable access to care. Kidney Int..

[2] Kostev K., Lang M., Trobs S.O., Urbisch S., Gabler M. (2025). The Underdiagnosis of Chronic Kidney Disease in Patients with a Documented Estimated Glomerular Filtration Rate and/or Urine Albumin-Creatinine Ratio in Germany. Medicina.

[3] Ostermann M., Lumlertgul N., Jeong R., See E., Joannidis M., James M. (2025). Acute kidney injury. Lancet.

[4] Jadoul M., Aoun M., Masimango Imani M. (2024). The major global burden of chronic kidney disease. Lancet Glob. Health.

[5] Liyanage L., Muzaale A., Henderson M. (2019). The true risk of living kidney donation. Curr. Opin. Organ Transplant..

[6] Haugen A.J., Hallan S., Langberg N.E., Dahle D.O., Pihlstrom H., Birkeland K.I., Reisaeter A.V., Midtvedt K., Hartmann A., Holdaas H. (2022). Increased risk of ischaemic heart disease after kidney donation. Nephrol. Dial. Transplant..

[7] Chawla L.S., Eggers P.W., Star R.A., Kimmel P.L. (2014). Acute kidney injury and chronic kidney disease as interconnected syndromes. N. Engl. J. Med..

[8] Yan L.J., Allen D.C. (2021). Cadmium-Induced Kidney Injury: Oxidative Damage as a Unifying Mechanism. Biomolecules.

[9] Scammell M.K., Sennett C.M., Petropoulos Z.E., Kamal J., Kaufman J.S. (2019). Environmental and Occupational Exposures in Kidney Disease. Semin. Nephrol..

[10] Eom S.Y., Yim D.H., Huang M., Park C.H., Kim G.B., Yu S.D., Choi B.S., Park J.D., Kim Y.D., Kim H. (2020). Copper-zinc imbalance induces kidney tubule damage and oxidative stress in a population exposed to chronic environmental cadmium. Int. Arch. Occup. Environ. Health.

